# Ventilator-induced diaphragm dysfunction: translational mechanisms lead to therapeutical alternatives in the critically ill

**DOI:** 10.1186/s40635-019-0259-9

**Published:** 2019-07-25

**Authors:** Oscar Peñuelas, Elena Keough, Lucía López-Rodríguez, Demetrio Carriedo, Gesly Gonçalves, Esther Barreiro, José Ángel Lorente

**Affiliations:** 1 Intensive Care Unit, Hospital Universitario de Getafe, Carretera de Toledo, km 12.5, 28905 Getafe, Madrid Spain; 2 Centro de Investigación en Red de Enfermedades Respiratorias [CIBERES], Instituto de Salud Carlos III [ISCIII], Madrid, Spain; 3 Pulmonology Department-Muscle Wasting and Cachexia in Chronic Respiratory Diseases and Lung Cancer Research Group, IMIM-Hospital del Mar, Parc de Salut Mar, Health and Experimental Sciences Department [CEXS], Barcelona, Spain; 4 Universitat Pompeu Fabra [UPF], Barcelona Biomedical Research Park [PRBB], Barcelona, Spain; 5 Universidad Europea, Madrid, Spain

**Keywords:** Diaphragm dysfunction, Mechanical ventilation, Critically ill patient, Diaphragmatic fatigue, Respiratory muscles, Weaning failure

## Abstract

Mechanical ventilation [MV] is a life-saving technique delivered to critically ill patients incapable of adequately ventilating and/or oxygenating due to respiratory or other disease processes. This necessarily invasive support however could potentially result in important iatrogenic complications. Even brief periods of MV may result in diaphragm weakness [i.e., ventilator-induced diaphragm dysfunction [VIDD]], which may be associated with difficulty weaning from the ventilator as well as mortality. This suggests that VIDD could potentially have a major impact on clinical practice through worse clinical outcomes and healthcare resource use. Recent translational investigations have identified that VIDD is mainly characterized by alterations resulting in a major decline of diaphragmatic contractile force together with atrophy of diaphragm muscle fibers. However, the signaling mechanisms responsible for VIDD have not been fully established. In this paper, we summarize the current understanding of the pathophysiological pathways underlying VIDD and highlight the diagnostic approach, as well as novel and experimental therapeutic options.

## Background

Invasive mechanical ventilation (MV) is a life-saving procedure applied to critically ill patients to achieve adequate pulmonary gas exchange and unload excessive respiratory muscle work. Observational studies report that the use of mechanical ventilation has led to a decrease in the mortality rate of critically ill patients [1–3].

Nevertheless, and based on the advances in the understanding that mechanical ventilation itself can cause and potentiate lung injury [4, 5], translational research has focused on ventilatory strategies and adjunctive measures aimed at mitigating this so-called ventilator-induced lung injury (VILI) [6].

Recently, a similar concern has emerged with regard to the potential adverse effects of invasive mechanical ventilation on the respiratory muscles. This entity was originally termed ventilator-induced diaphragmatic dysfunction (VIDD) [7], although it may also involve other respiratory muscles. Both animal [8, 9] and recent human [10, 11] studies have demonstrated that complete diaphragm muscle unloading or inactivity during invasive MV induces a rapid and profound loss of diaphragm muscle force-generating capacity. In 2008, it was [12] demonstrated that VIDD occurs in critically ill patients and is characterized by marked diaphragm atrophy of both slow-twitch and fast-twitch fibers, with evidence of oxidative stress in addition to proteolysis.

It is well known that muscle dysfunction in critically ill patients defined as ICU-acquired weakness is associated with weaning failure and unfavorable outcomes in critically ill patients [13, 14]. However, it remains unclear whether the specific changes in the diaphragm caused by mechanical ventilation significantly impact clinical outcomes. A recent study [15] confirmed the results of prospective studies that associated VIDD with difficulty weaning and poor clinical outcomes. The authors also found that the development of a decreased diaphragm thickness was associated with a lower daily probability of liberation from ventilation adjusted hazard ratio (0.69; 95% confidence interval (CI), 0.54–0.87; per 10% decrease), prolonged ICU admission (adjusted duration ratio, 1.71; 95% CI, 1.29–2.27), and a higher risk of complications (adjusted odds ratio, 3.00; 95% CI, 1.34–6.72).

Currently, VIDD represents one of the most challenging fields within translational research applied to critically ill patients. This review provides the knowledge necessary for intensivists to better understand the signaling mechanisms responsible for VIDD, as well as summarizes the development of diagnostic bedside tools. Based on these findings, future therapeutical approaches may have the ability to prevent VIDD and to improve weaning success as well as other relevant clinical outcomes.

## Epidemiology of diaphragm dysfunction in mechanically ventilated patients

Multiple recent studies have shown that VIDD is reported in up to 53% of mechanically ventilated patients within 24 h of intubation. An additional 26% may develop VIDD while on mechanical ventilation during their stay in the intensive care unit (ICU).

In the literature, the variations observed in the incidence of this complication are based fundamentally on the diagnostic tool used. The assessment of the transdiaphragmatic twitch pressure (PdiTw) generated in response to bilateral anterior magnetic phrenic nerve stimulation (BAMPS) is an objective, nonvolitional measurement which represents the gold standard technique used to specifically assess diaphragm strength. According to several observational studies, mechanically ventilated patients in the ICU, on average, generate a PdiTw that is only 20% of normal [16]. The use of ultrasonography as a diagnostic tool is easier in everyday practice when compared to performing a PdiTw, and it is becoming an increasingly popular alternative method for the diagnosis of VIDD [17]. Additional studies have confirmed that around 60 to 80% of mechanically ventilated patients manifest clinically significant diaphragm dysfunction as evaluated by bedside diaphragmatic ultrasound [18–20]. Overall, a recent study indicates that diaphragm weakness is present twice as often as limb weakness in critically ill patients [21, 22].

The potential improvement in the outcome that might be obtained by implementing prevention or therapeutic strategies for diaphragm atrophy caused by ventilation is therefore also uncertain.

## Pathogenic mechanisms underlying ventilator-induced diaphragm dysfunction

Most studies show that VIDD occurs in a progressive, time-dependent manner [11], although its degree is influenced by different MV modes and other clinical variables [23, 24].

**Fig. 1:**
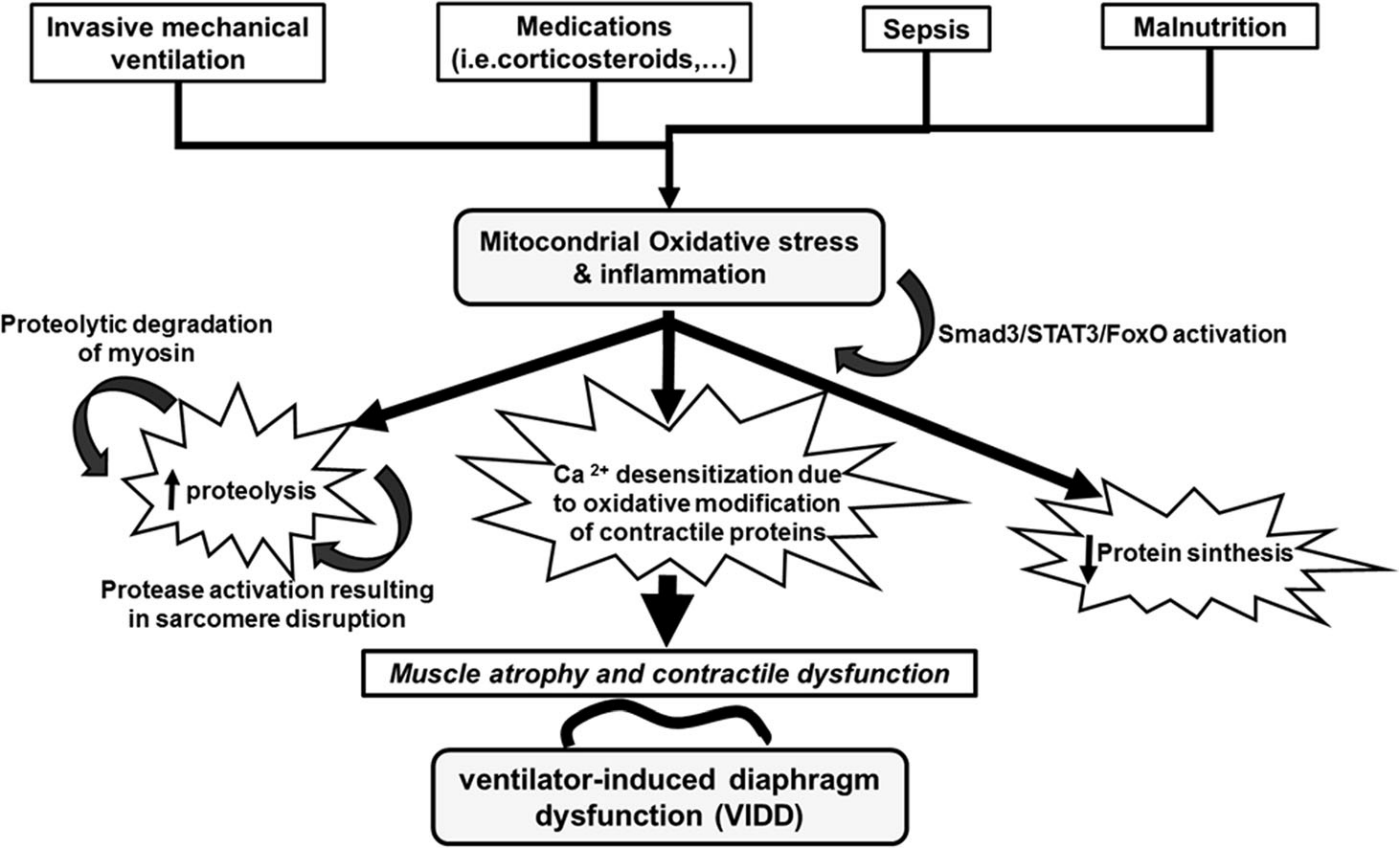
Summary of the current understanding of the molecular pathways contributing to ventilator-induced diaphragm dysfunction (VIDD) in critically ill patients. As shown, different conditions can lead to diaphragm atrophy via an imbalance between proteolysis and protein synthesis [11, 14], whereas remaining muscle proteins may be impaired by enhanced oxidation and dephosphorylation [15–17]. Inflammation and oxidative stress are proposed to be the major drivers of these impairments [17]. In addition, certain drugs can impair neural drive and excitation-contraction coupling

The molecular mechanisms underlying VIDD currently represents one of the more attractive fields of translational research in critically ill patients. We summarize some of the key discoveries that may provide a unified mechanistic framework for understanding the pathogenesis of VIDD (Fig. 1).

The mechanical properties of diaphragm muscle fibers are characterized by the relationship between cytosolic-free calcium, cross-bridge attachment/cross-bridge cycling rate, and sarcomere length [25]. Any factor that influences one or more of these variables can impact diaphragm muscle force generation.

Nonetheless, it seems likely that the cause of MV-induced diaphragm contractile dysfunction is multiplicative and includes oxidative modifications to contractile proteins resulting in depressed fiber sensitivity to calcium, protease activation leading to sarcomere disruption, and a loss of myosin heavy chain protein.

### Mitochondrial oxidative stress (MOS)

Overall, we can consider that VIDD is characterized by a disbalance in protein homeostasis defined as a decrease in diaphragm protein synthesis and an increase in diaphragm protein degradation. The activation of catabolic pathways, induced in the diaphragm under mechanical ventilation, occurs in the short-term under controlled modes of mechanical ventilation [26]. This appears to be related to an upregulation of the oxidative stress-mediated ubiquitin–proteasome pathway that would lead to an increase in protein degradation and thus atrophy.

Therefore, mitochondrial oxidative stress (MOS) may play an integral role in MV-induced atrophy of the diaphragm by inducing catabolism [27, 28].

The force component of VIDD can also be affected by MOS in the following two ways: firstly, by producing free radicals that can induce a post-translational modification of muscle proteins [e.g., oxidation] and, hence, alter their structure and function, specifically reducing the calcium sensitivity of myofilaments [29], and secondly, MOS can cause a metabolic switch, by reducing mitochondrial oxidative phosphorylation and increasing glycolysis [30]. The lipid accumulation seen in diaphragms under mechanical ventilation [31] could trigger important metabolic pathways as an accelerated glycolysis. This allows for more intermediate metabolites to be converted into fatty acids and so the decreased breakdown of fatty acids due to altered mitochondrial function. In fact, lipotoxicity could serve as a stimulus to VIDD, but further studies are required to determine if these are the triggers or the consequence of MOS.

### Autophagy

Autophagy is a catabolic process that degrades cytosolic proteins and organelles using lysosomal proteases. More specifically, lysosomal proteases (i.e., cathepsins) have the biological function of removing both organelles and nonmyofibril cytosolic protein aggregates [32]. During autophagy, targeted cytosolic components are encapsulated into a double-membrane vacuole called the autophagosome. When complete, the autophagosome moves through the cytosol to the lysosome where the contents of the autophagosome are degraded.

A recent study found that prolonged mechanical ventilation increases the expression of key autophagy proteins [e.g., ATG5, ATG7, and beclin 1] and the number of autophagosomes in the human diaphragm [33]. Furthermore, emerging evidence reveals an increase in autophagy biomarkers in rodent diaphragms following 8–18 h of controlled mechanical ventilation [34, 35].

However, it is not clear whether autophagy is an effector of VIDD, or a protective mechanism that may actually offset VIDD [36, 37]. Critically ill patients have been shown to have both impaired autophagic vacuole formation and mitophagy in their skeletal muscles, resulting in a reduced clearance of proteins that result from cellular damage [38]. On the other hand, several experimental studies have shown a reduction of VIDD by enhanced autophagic pathways. These include fragmentation of intermyofibrillar mitochondria as an early event in a mouse model of VIDD [39], and stimulation of autophagy with n-acetylcysteine or rapamycin [40]. Mechanical ventilation-associated increases in autophagy may actually help to clear cells of dysfunctional mitochondria and thereby improve muscle function, as has been shown to be the case in other murine models of muscle disease [41].

### Calpains

Calpains are cysteine proteases that promote muscle atrophy by cleaving over 100 different cellular proteins, and the two ubiquitous calpains located in skeletal muscle are calpain I and II [42]. Numerous studies reveal that calpain cleavage of Z line-associated proteins (i.e., titin and nebulin) is responsible for the release of myofilament proteins that are then degraded by the ubiquitin-proteasome system and perhaps other proteases as well [37, 43].

Importantly, it has been established that prolonged mechanical ventilation activates calpain in the diaphragms of both humans and animals [44]. In fact, recent experimental studies revealed that pharmacological inhibition of calpain activation could partially protect against VIDD [45].

Specific diaphragm muscle proteins have been identified that are degraded during mechanical ventilation-induced sarcomere damage, concretely both titin and myosin. A recent work found a significant reduction in rat diaphragm single muscle fiber force production after 18 h of mechanical ventilation, which was accompanied by a proportional loss of myosin heavy chain concentration together with a titin dysfunction in diaphragm fibers [46]. Interestingly, these findings were not found in other peripheral muscles.

Another possible link between mechanical ventilation-induced oxidative stress and diaphragm contractile dysfunction is the connection between reactive oxygen species (ROS) and calpain activation. MV-induced ROS production in diaphragm muscle promotes calpain activation [47, 48]; in turn, active calpain can degrade key cytoskeletal proteins involved in maintaining sarcomere structure. This results in sarcomere disruption, which impairs the muscle’s ability to generate force.

Finally, recent studies have shown that regulatory cross-talk exists between calpain and caspase-3 in the diaphragm during controlled MV in rats. Caspase-3 belongs to a large family of cysteine proteases and plays an important role in apoptosis. This protease may also contribute to muscle protein degradation during a variety of muscle wasting conditions.

There is a body of research demonstrating that full support MV activates caspase-3 in both the rodent and human diaphragm and induces myonuclear apoptosis in the diaphragm [25, 44, 49, 50].

The ubiquitin-proteasome system of proteolysis plays an important role in muscle protein catabolic pathways during a variety of wasting conditions [51]. Recent experimental studies have demonstrated that prolonged and controlled mechanical ventilation may increase the activation of the ubiquitin-proteasome system of proteolysis in both human and rat diaphragms [52, 53]. Studies on the effects of partial support or assisted mechanical ventilation on protease activation in humans remain unpublished.

### Ventilator-induced impairment of diaphragmatic contractile function

Both experimental and human studies have shown that prolonged mechanical ventilation induces diaphragm contractile dysfunction [11]. In an animal study, the investigators found a depression of in vivo diaphragm contractility at 5 days of mechanical ventilation in piglets [54]. Several experimental studies consistently report that invasive mechanical ventilation results in a rapid and time-dependent decrease in diaphragmatic force production measured in vitro through the electrical stimulation of the diaphragm [55–58]. A recent murine model showed diaphragm contractile dysfunction after 6 h of ventilatory support [59]. The ventilator-induced diaphragmatic contractile dysfunction in animals may be directly associated to diaphragm contractile inactivity since partial ventilator modes or short periods of intermittent spontaneous breathing can reduce the magnitude of full ventilator support-induced contractile dysfunction [9, 36]. In this respect, there are few experimental studies focused on the impact of prolonged mechanical ventilation on diaphragm weakness in animals [60–64].

## Risk factors for acquiring VIDD

In critically ill patients, some factors have been identified that along with prolonged mechanical ventilation adversely impact diaphragm contractile function including senescence [65, 66], intravenous medications such as neuromuscular blockers [67], and/or glucocorticoids [68].

These studies however present divergent findings, and further research is needed to address the effect of treatments on diaphragm function in animals using both partial and controlled mechanical ventilation.

Although human information is limited, age-related respiratory muscle dysfunction resulting in a decrease in strength has been demonstrated, with the fall of 0.8 to 2.7 cm H_2_O per year from the maximal inspiratory pressure (MIP) between the ages of 65 and 85 years [67]. A 25% drop in transdiaphragmatic pressure in adults between 65 and 75 years of age has also been described [69, 70].

One mechanism that explains these changes is the cumulative effect of active oxygen radicals, which can trigger proteolytic processes. It has also been suggested that with increasing age, there is a remodeling of the muscle fibers in which the fast myosin fibers are replaced by slow type isoforms [71].

The mechanisms involved in the deleterious effect of mechanical ventilation on diaphragmatic dysfunction are not fully elucidated. Recently, a translational study found that invasive mechanical ventilation with positive end-expiratory pressure (PEEP) results in longitudinal atrophy of the diaphragm fibers that is modulated by the elasticity of the titin of giant sarcomeric protein [72].

Hypercapnia, on the other hand, is more clearly protective against VIDD. In this regard, piglets ventilated with increased dead space to achieve moderate hypercapnic acidosis [PaCO_2_ 55–70 mmHg] showed preserved diaphragmatic force production after 72 h of mechanical ventilation [73]. A similar level of hypercapnia induced by adding inspired CO_2_ also attenuated several aspects of VIDD in rats [74].

The cellular mechanisms underlying the improvements associated with hypercapnia are yet to be fully understood but likely involve anti-inflammatory and/or antioxidant effects. VIDD however does not appear to be correlated with ventilator-induced lung injury [60, 75, 76].

The majority of VIDD seen in critically ill patients, however, does not appear to be the consequence of any easily treatable conditions. In many cases, the potential mechanisms underlying diaphragmatic injury are induced by mechanical ventilation. There is also strong evidence that processes other than VIDD, including sepsis and other systemic infections, are responsible for various forms of diaphragmatic myotrauma.

## Diagnostic approach for VIDD

### Tests of respiratory muscle strength

Accurate evaluation of diaphragmatic contractile function in the setting of critically ill patients undergoing mechanical ventilation continues to be difficult due to multiple factors.

These include limitations in the techniques used, the volitional aspect of some maneuvers of diaphragmatic contraction, the interference of positive pressure ventilation, and inter-observer heterogeneity [77]. Briefly, the tests most used in assessing respiratory muscle endurance are summarized in Table 1.

**Table 1:** Summary of the clinical tests used in the assessment of respiratory muscle strength [63]

	Test	Threshold values	Advantages	Disadvantages
Volitional Tests	Maximum static inspiratory pressure (PImax)	Male < − 45 cm H_2_O Female < − 30 cm H_2_O	Easy to perform. Normal values are available	Difficult interpretation. Lack of specificity
	Maximum static transdiaphragmatic pressure (PI,di,max)	Male < 40 cm H_2_O (2) Female < 30 cm H2O	Easy to perform. Well tolerated by patients.	Wide normal range. Limited usefulness in clinical practice. Limited normal data
	Sniff transdiaphragmatic pressure (Sniff Pdi)	Male < 100 cm H_2_O (2) Female < 70 cm H_2_O	Requires little practice. It is relatively reproducible. Range of normal values	Technical limitations Variability.
	Maximum sniff pressures (nasal)	Male < 50 cm H_2_O Female < 45 cm H_2_O		
	Maximum cough pressure	Male < 130 cm H_2_O Female < 95 cm H_2_O	Normal ranges available	Limited validation in critically ill patient.
Nonvolitional tests	Twitch transdiaphragmatic pressure (PdiTw)	Male and female < 18 cm H_2_O	Measurement specific for the diaphragm and is not influenced by the central nervous system	Requires considerable skill. Uncomfortable for patients
	Diaphragm excursion (DE)	< 10 cm	Provides both morphological and functional information in real time. Allows repeated measurements over time and monitoring recovery	Learning curve. Inter-observer variations. Availability. Reproducibility
	Inspiratory diaphragm thickening fraction (TFdi)	< 20%		

The maximum static inspiratory pressure (PImax) in ventilated patients has commonly been used, but its interpretation is effort-dependent. It represents the combined action of all inspiratory muscles rather than isolating the diaphragmatic contraction, can be affected by underlying lung diseases, and is associated with widely variable predicted values [78, 79]. A PImax of − 80 cm H_2_O usually excludes clinically important inspiratory muscle weakness [77, 78], and bilateral diaphragm paralysis can be expected to decrease PImax < 30% of the predicted values [79].

The transdiaphragmatic pressure [PTIdi] can also be measured by simultaneously recording the esophagus and the stomach pressures, but the interpretation of the results is also limited and may be dependent on the level of patient co-operation, the depth of sedation, or the use of neuromuscular blockers.

Other non-invasive beside volitional tests such as the sniff maneuvers can be used in combination to enhance the performance of certain tests.

Specifically, *sniff nasal pressure* (Sniff Pnasal) may result as precise as when more invasive tests (i.e., sniff esophageal pressure) are added to the evaluation [80–82]. The assessment of a diaphragmatic contraction following phrenic nerve stimulation by electric or magnetic twitch performed during phrenic nerve conduction studies is the accepted standard non-volitional method to quantify the mechanical function of the diaphragm. During these evaluations, the twitch transdiaphragmatic pressure (Twitch Pdi) is used, and a cutoff value lower than 18 cm H_2_O is highly suggestive of diaphragmatic weakness [83].

However, these techniques depend to a great extent on the effort of the patient in addition to other factors. For instance, these maneuvers require experience and specialized equipment as well as access to this equipment in routine ICU clinical practice. The capacity to predict clinical outcomes, such as weaning failure, with the use of any of the abovementioned tests of inspiratory, specific diaphragm, or expiratory muscle strength, is broadly similar to any other test [84].

Nevertheless, a combination of tests can substantially increase the diagnostic accuracy [85–88].

Diaphragmatic contraction can be assessed using magnetic resonance imaging and fluoroscopy, but the problems associated with ionizing radiation, patient transport, and high cost are factors limiting daily bedside use [89, 90].

### Ultrasonography

In recent years, diaphragmatic ultrasound has become the most useful bedside for the clinician to identify patients with VIDD subjected to invasive mechanical ventilation.

This is most likely because it has been shown to be safe and easy to perform, while allowing both morphologic assessment and functional evaluation of the muscle with high inter-observer agreement [91, 92]. It is also possible to perform a follow-up of the initial clinical evaluation, providing information on time course and recovery.

There are two main ultrasonography parameters used to assess diaphragmatic function. These include the dynamic evaluation of diaphragmatic excursion (DE) and the inspiratory diaphragm thickening fraction (TFdi). Diaphragmatic excursion (DE) can be easily measured with a 3–5-MHz probe in M-mode and represents the mobility of the diaphragm in inspiration and expiration during a spontaneous mode of mechanical ventilation. However, DE depends on the amount of ventilator support and PEEP; accordingly, a recent study indicated that DE should not be used to assess diaphragmatic contractility in patients receiving mechanical ventilation [93, 94].

The second parameter, TFdi, measures muscle thickening in the zone of apposition of the diaphragm to the rib cage with a 10–15 MHz probe in B or M mode.

**Fig. 2:**
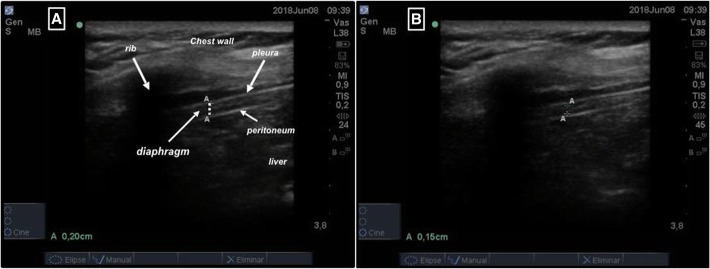
Representative ultrasound image at the zone of apposition in B-mode view of the diaphragm during inspiration (**a**) and expiration (**b**). The diaphragm is identified as a 3-layer comprising two hyperechoic lines representing the pleural and peritoneal membranes and a middle hypoechoic layer representing the diaphragmatic muscle itself (*with permission of the Intensive Care Unit from the Hospital Universitario de Getafe)*

The transducer is placed in the same way as for the measurement of the diaphragmatic excursion. Following placement, measurements of the diaphragmatic thickness in both inspiration and expiration are taken (Fig. 2).

TFdi is defined as [[thickness at end-inspiration − thickness at end-expiration]/thickness at end-expiration]*.* The most accepted cutoff values are 10–14 mm for DE and 30–36% for TFdi [95].

Both DE and TFdi have been shown to correlate with functional measurements of diaphragmatic function in spontaneously breathing patients, but the use of ultrasound in the process of weaning remains controversial [96, 97].

Several clinical studies suggest that these ultrasound parameters can be reliable predictors of weaning and extubation outcomes. Indeed, a recent study [98] found a prevalence of VIDD of 29% (by DE < 10 mm or paradoxical movements of the diaphragm). Additionally, patients diagnosed with VIDD had a longer duration of mechanical ventilation.

On the other hand, a study [96] reported in 63 adult critically ill patients with mechanical ventilation that a TFdi ≥ 30% presented a sensitivity of 88% and a specificity of 71% for extubation success, with a positive predictive value of 91% and a negative value of 63%, with an area under the curve of 0.79. In combination, these two methods of assessment of diaphragmatic function during spontaneous breathing are useful tools for the determination of patients at risk of invasive mechanical ventilation withdrawal failure.

Recently, a systematic review that included 13 studies on 742 adult critically ill patients found that ultrasound had excellent intra- and inter-observer reproducibility, as well as higher accuracy in investigating diaphragm dysfunction compared with other weaning indexes or fluoroscopy [99]. Finally, another metaanalysis that included nineteen studies (1071 participants) found that ultrasound may help predict weaning outcome, but its accuracy and interpretation involve several pitfalls [100]. Therefore, ultrasound is emerging as a new noninvasive tool used to monitor respiratory workload during assisted mechanical ventilation and to assess the progression of VIDD. Nevertheless, further randomized clinical trials are needed to determine whether ultrasound could guide clinical decisions for improving outcomes in critically ill patients.

A novel field of research is the implementation of tissue Doppler as a diagnostic tool in the analysis of diaphragm function. Tissue Doppler imaging (PW-TDI, pulse wave tissue Doppler imaging) is an echocardiographic technique and promises a number of applications to further extend the unique diagnostic role in echocardiography. In particular relation to the diaphragm, it might allow the assessment of regional diaphragmatic contractile function at rest and with stress. It should also provide a reliable identification of viable respiratory contractile muscle. There is currently a French clinical trial which aims to look for correlations between the time movement mode and tissue Doppler in the assessment of a threshold value of diaphragm mobility in patients in the postoperative period following scheduled cardiac surgery (ClinicalTrials.gov Identifier: NCT03295344).

Diaphragm electromyography (EMGdi) reflects the muscle electrical activity, and it is considered the gold standard in the assessment of neural respiratory drive.

Electromyography (EMG) comprises the temporal and spatial summation of neural impulses from the brain that are translated into muscle fiber action potentials. There are different approaches to obtaining a diaphragm EMG, and at the bedside, it can be recorded using an esophageal catheter with multiple electrodes [101].

The processed signal can be obtained easily and continuously in ICU patients and is referred to as the amplitude of the electrical activity of the diaphragm (EAdi). EAdi records action potentials from the diaphragm and can therefore assess whether the phrenic nerve is intact. This may be helpful in monitoring respiratory muscle loading, patient–ventilator synchrony, and efficiency of breathing in critically ill patients [102]. EAdi is tightly correlated to a patient’s inspiratory effort and is a good estimate of diaphragm function. Combining EAdi with the breathing pattern provides indices that evaluate the diaphragm’s contribution to the generation of tidal volume (VT). For example, the ratio of VT to EAdi (VT/EAdi) represents the neuroventilatory efficiency of the diaphragm.

This index reflects the ability of the diaphragm to convert the respiratory drive into ventilation. A low VT/EAdi suggests severe impairment of neuromechanical coupling. EAdi-derived indices have been used to identify patients with weaning failure [103]. Interestingly, monitoring neuroventilatory efficiency during a SBT enables very early detection of patients likely to fail the test. However, the performance of EAdi-derived indices to predict weaning failure is not better than the performance of other clinical, non-invasive, and bedside weaning tests.

Similarly, the electrical activity of the diaphragm (EAdi) records action potentials from the diaphragm and therefore assesses whether the phrenic nerve is intact [104].

A clinical study [105] reported that the pressure developed by the respiratory muscles (Pmusc) is related to the electrical activity of the diaphragm (EAdi) and that the ratio of Pmusc/EAdi could estimate inspiratory effort. However, a study published by the same author found no correlation between the Pmusc/EAdi ratio and ventilator variables [106].

The second tool is the diaphragm electromyography (EMGdi), which measures the activation of action potentials along the diaphragm muscle and can be used to assess muscle contractility. Intramuscular electrodes can be used to record, relatively selectively, from the diaphragm and intercostal muscles [107].

However, the techniques are invasive and technically difficult.

There is a body of research showing that EMGdi is feasible in routine clinical care for monitoring respiratory muscle function. However, there are some limitations in the interpretation of EAdi: it is still necessary to identify the threshold level of EAdi that should be achieved in the daily management of mechanically ventilated patients [108]; respiratory muscle activity may be suppressed by sedatives; the insertion of an esophageal catheter carries a low complication risk, but it is an uncomfortable and invasive procedure in non-sedated patients.

Nevertheless, there are nasogastric tubes for enteral feeding, which are already commercially available, with EMG electrodes.

Taken together, EAdi is a well-evaluated parameter to monitor respiratory muscle unloading and patient–ventilator synchrony from an early phase of critical illness. The implementation of EAdi would be clinically relevant as an important tool for respiratory muscle monitoring during mechanical ventilation and weaning.

## Potential therapeutical approaches for VIDD

One of the challenges in translational research is the development of a management strategy in order to avoid VIDD in the critically ill patient, with the aim of improving clinical outcomes.

No preventive or therapeutic interventions have been tested in clinical trials.

Potential treatments arising from translational or from small clinical studies should be considered as initial steps towards large clinical trials. These larger trials are necessary in order to establish a definitive approach to VIDD in the critically ill patient. The current strategies which target preventive, experimental, and/or therapeutic approaches might support the development of well-designed clinical trials. Any VIDD management strategies should firstly consider the optimal medical treatment of concurrent conditions, such as malnutrition [109], in addition to any other associated factors such as sepsis [110].

### Experimental drugs

A better understanding of the pathophysiological mechanisms involved in VIDD at the cellular, transcriptional, and molecular levels is leading the search for new therapeutic approaches. This includes experimental pharmacological agents such as the use of mitochondria-targeted antioxidants [111], JAK-STAT inhibitors [112, 113], different modulators of proteolysis pathways including autophagy [40], and myofilament calcium sensitizers [114]. Future studies should be focused at translating these findings into the clinical setting in critically ill patients.

Transcription factors of the FOXO and signal transducer and activator of transcription (STAT) families are upregulated and act as important mediators of VIDD [115].

Data from both animal [28, 116, 117] and human studies [118] have highlighted that the main source of oxidative stress in the diaphragm during mechanical ventilation is excess mitochondrial reactive oxygen species (ROS) production. However, the mechanisms triggered by invasive mechanical ventilation leading to the development of mitochondria-derived oxidative stress are poorly understood.

An experimental study found in a rat model of mechanical ventilation that the intravenous infusion of an antioxidant (Trolox, an analog of vitamin E with antioxidant activity) prevented VIDD by attenuating the oxidative stress and subsequent proteolysis and contractile dysfunction during mechanical ventilation [119].

In summary, antioxidant agents acting on more than one molecular target may mediate VIDD produced by oxidative stress, and therefore, this represents an important clinical issue that warrants further investigation.

### Preventives

Since VIDD has been identified as a cause of weaning failure, improving respiratory muscle function is an important strategic option. In the ICU, the most promising therapy seems to be inspiratory muscle training (IMT); however, only a few studies have been conducted. When looking at studies on inspiratory muscle training, two factors must be considered. First, the modalities of the “control” arm to which inspiratory muscle training is compared should be carefully examined because there is considerable heterogeneity in practice. Second, the impact of the intervention should have clinical relevance. There is no real benefit of improving inspiratory muscle force itself; attention has to be paid to clinical outcomes, such as shortened duration of mechanical ventilation, successful extubation, or greater survival. The largest study enrolled 69 long-term ventilated patients and randomized them to receive either IMT (defined as a 5-day-a-week program with four sets breathing through a threshold inspiratory muscle trainer of 6 to 10 breaths per day) or control training [118]. Patients in the control group used a resistive inspiratory muscle training device set at the largest opening. In this study, IMT significantly improved maximal inspiratory pressure and successful weaning was more likely. A randomized study [119] performed on ventilated patients allocated them to either inspiratory strength training in addition to usual care or usual care only. Each training session consisted of five sets of loaded breaths [40% maximum inspiratory pressure], twice a day, 7 days a week.

In this study, IMT significantly increased maximum inspiratory pressure but did not affect clinical outcomes such as weaning time. In another study, it was recently reported that IMT performed after successful extubation improved respiratory muscle function, but without any clinical benefit [120]. In conclusion, IMT appears to be effective by improving markers of respiratory muscle function but fails to produce any significant clinical outcomes, in particular on the duration of mechanical ventilation.

Therefore, IMT is feasible and appears safe in critically ill patients with respiratory muscle weakness and weaning failure [121]. Studies in other patient categories, including COPD, indicate that IMT improves outcome. In our opinion, IMT can be included as an endurance training strategy in stable, difficult-to-wean patients with confirmed VIDD. Further studies are needed to determine the optimal training protocol and appropriate timing for initiation of IMT.

When choosing ventilatory modes, maintaining spontaneous breathing efforts during invasive mechanical ventilation seems protective and seems to alleviate VIDD in healthy animals [122, 123]. Early in acute critical illness, modes of controlled mechanical ventilation may often be required, but an early switch to an assisted mode seems clearly desirable [124]. A recent study [125] found that the daily reductions in thickness were 7.5% during controlled mechanical ventilation, 5.3% during high pressure support ventilation, and 1.5% during low pressure support ventilation, whereas the diaphragm thickness increased 2.3% under conditions of spontaneous breathing and CPAP. In another large observational and prospective study [126], diaphragm thickness was assessed in 107 critically ill patients during the first week of invasive mechanical ventilation. The authors found a rapid decrease in more than 10% of diaphragm thickness during the first several days of mechanical ventilation in 44% of subjects. The factors significantly associated with higher contractile activity were the use of lower ventilator driving pressures and partially assisted modes of ventilation (*p* value 0.01 and 0.02, respectively).

The preferred ventilation mode, the optimal level of support (“unloading”), or the rate of support reduction for patients is currently unknown. Spontaneous breathing is well maintained with so-called effort-adapted modes [127], for example neurally adjusted ventilatory assist (NAVA) or adaptive support ventilation (ASV). ASV seems to mitigate deleterious effects of mechanical ventilation on the diaphragm of piglets [128–130]. NAVA provides support in unison with the patient’s inspiratory neural effort [131]. As the support level is titrated against the patient’s respiratory demand, patients are protected against over-assist [132, 133]. This approach was successfully used in various clinical ICU settings, especially in patients with critical illness myoneuropathy [134, 135].

### Pharmacological agents

Methylxanthines, especially aminophylline and theophylline, have been widely prescribed in poorly controlled asthma or COPD based on some of their pathophysiological mechanisms of action. These drugs mainly relax airway smooth muscle by inhibiting phosphodiesterase-3 activity, leading to bronchodilation [136]. They antagonize adenosine A1 and A2 receptors, which also results in bronchodilation; they act as anti-inflammatory agents increasing the effect of interleukin-10 and preventing the translocation of the proinflammatory transcription factor nuclear factor-B [137]. Theophylline also enhances histone deacetylase-2 activity, which is reduced by oxidative stress.

The increased histone deacetylase-2 activity reduces formation of peroxynitrite radicals, stimulates the respiratory neuronal network, and increases the activity of respiratory muscles, including the intercostal and transversus abdominis muscles, as well as the diaphragm [138–142].

These findings suggest that methylxanthines may have a role as therapeutic agents in ventilated patients with weaning difficulties associated with VIDD [143–146].

A study using phrenic nerve conduction showed that theophylline infusion rapidly reversed the reduction of transdiaphragmatic pressure resulting from resistive loaded breathing in normal human subjects [147].

In subjects with severe COPD, theophylline significantly increased the maximal transdiaphragmatic pressure and suppressed diaphragmatic fatigue when compared with placebo [148]. Several studies have shown the favorable effects of theophylline on human respiratory muscle function, and the drug is commonly used in patients weaning from mechanical ventilation. However, few studies have reported clinical experience with theophylline with regard to clinical outcomes such as weaning from mechanical ventilation. Recently, a clinical trial found that low-dose (median, 200 mg/d intravenously) theophylline treatment significantly improved the diaphragmatic movements in ventilated patients with evidence of VIDD [149]. In this study, no significant adverse effects were reported. This effect may be related to the relatively low serum concentration [mean 4.6 mg/L on day 3] of theophylline compared with studies that used higher doses of theophylline (mean serum concentration ≥ 10 mg/L) due to a proven pathophysiological effect even at these low concentrations. Another study analysis from a Taiwanese group included 160 patients that required invasive mechanical ventilation longer than 21 days and compared retrospectively 84 patients that received aminophylline (200 mg/twice a day) with 76 patients included in the nontheophylline group. The mean treatment duration was 23 [9–34] days, and the mean serum concentration of theophylline after the 3rd day of treatment, which was checked in 49 (58%) of the 84 patients, was 9.3 ± 3.3 mg/ml [150].

The investigators found that the primary outcome (PImax) was significantly better in the theophylline group than in the nontheophylline group (30.1 ± 9.7 cm H_2_O vs. 26.9 ± 9.1 cm H_2_O; *p* value = 0.034), but no statistically significant differences in clinical outcomes were found (successful weaning in the aminophylline group 66/84, 79%, versus 50/76, 66%, in the control group, *p* = 0.071; duration of mechanical ventilation in the aminophylline group mean 20 ± 14 days, 79%, versus mean 23 ± 14.5 days, in the control group, *p* = 0.192), and with no significant adverse reactions among the study patients.

The finding that low-dose theophylline significantly improved VIDD is encouraging, considering that the drug is often withheld due to concerns over its adverse effects, and therefore, further large randomized clinical trials are needed to implement this pharmacological approach in the management of VIDD.

Although the ultimate pathophysiological mechanisms are not as yet fully understood, changes in intracellular calcium level also appear to be an important basis for VIDD. In a murine model of VIDD, a rapid remodeling of the sarcoplasmic reticulum (SR) Ca [2+] release channel/ryanodine receptor (RyR1) in the diaphragm has been found [151]. Calcium sensitizers have been developed to treat similar pathology in cardiac muscle [152]. Indeed, levosimendan is the only calcium sensitizer approved for the treatment of heart failure in patients.

Experimental studies have shown that levosimendan improves calcium sensitivity of diaphragm muscle fibers in patients with COPD [153]. In healthy subjects, levosimendan reverses diaphragm fatigue and improves neuromechanical efficiency of the diaphragm [154]. In an experimental model of endotoxemia and mechanical ventilation, levosimendan decreased markers of oxidative and nitrosative stress in the diaphragm of endotoxemic mechanically ventilated mice, but did not attenuate diaphragmatic and systemic inflammatory responses [155].

However, in a recent trial [156] in which levosimendan was tested to prevent acute organ dysfunction in sepsis, patients who received the drug were more likely to fail weaning from mechanical ventilation [95%CI 0.60 to 0.97, *p* = 0.03].

Further studies are therefore needed to elucidate the potential indication of levosimendan in patients with diaphragm dysfunction.

Finally, a randomized clinical trial [ClinicalTrials.gov identifier NCT01721434] is currently investigating whether levosimendan facilitates liberation from the ventilator in patients difficult to wean from the ventilator.

In contrast to levosimendan, the effectiveness of other calcium sensitizers has so far only been studied in vitro. For example, exposure to EMD 57033 [a troponin activator] partially restored calcium sensitivity in diaphragm fibers isolated from piglets after 5 days of mechanical ventilation [157]. Taken together, calcium sensitizers might trigger a beneficial effect on diaphragm work [158]. However, further clinical trials are needed to prove the benefits of these calcium sensitizers in ICU patients with respiratory muscle weakness, and they are currently not recommended for the management of difficult-to-wean patients.

Pharmacological interventions aimed at restoring protein balance during muscle dysfunction in the critically ill patient seem a consistent pathophysiological approach. The most important endogenous anabolic hormones are growth hormone, insulin-like growth factor-1, insulin, and the anabolic steroid testosterone and its analogues. Although several trials have been conducted in patients with chronic disease, no clear clinical benefits have been identified and they are not indicated in ICU patients [159, 160].

### Non-pharmacological alternatives

It is well known that invasive mechanical ventilation may suppress the electrical activity of disused diaphragm muscle [9, 161]. Indeed, mechanical ventilation in a fully controlled support mode can abolish electrical activity in the diaphragm [56]. Even with an intact phrenic nerve function, there is no diaphragmatic stimulation during mechanical ventilation. A clinical study [56] found that 3 days of controlled mechanical ventilation (CMV) in the rabbit diaphragm completely inhibited the electromyogram signal compared with those animals randomized to continuous positive airway pressure (CPAP) for 3 days. In the rabbits that received CMV, there was accompanying histological changes in the diaphragm consisting of myofibril injury, findings which were not observed in the soleus muscle of the same animals.

Similar findings have been consistently demonstrated in different studies in rat [59], piglet [162], and both healthy humans and patients [163, 164]. As a result of these findings, an attractive hypothesis emerged some years ago that electrically pacing the diaphragm could prevent VIDD [165, 166].

Diaphragm pacing can be applied through a transvenous phrenic nerve pacing system designed for percutaneous placement into the left subclavian vein. In an experimental study, pigs subjected to mechanical ventilation that received transvenous phrenic nerve pacing in synchrony with ventilation exhibited less diaphragm atrophy [167]. Identifying the population who would benefit from this strategy will be the next clinical challenge. Surgically implanted systems for pacing the phrenic nerves and diaphragm have been used for the last 40 years in more than 2500 adult and pediatric patients with high-level spinal cord injury [167] and in patients with late-stage amyotrophic lateral sclerosis. Implanted systems are not suitable for critically ill patients because of the invasive and complex surgical procedure.

Brief transcutaneous stimulation of phrenic nerves is often used for diagnostic purposes, but is not suitable for therapeutic purposes. Critically ill patients requiring prolonged mechanical ventilation are not ideal candidates for surgically implanted systems, but may benefit from short-term phrenic nerve pacing to diminish VIDD.

Transvenous unilateral phrenic nerve stimulation has been shown to be safe in the treatment of central sleep apnea [168]. Another option is intermittent magnetic stimulation of the phrenic nerves, which is painless and noninvasive [169]. In rats, bilateral phrenic nerve stimulation preserved diaphragmatic force production after 18 h of mechanical ventilation [170]. A pilot study of unilateral phrenic nerve stimulation in sheep showed reduced atrophy and muscle fiber injury in the stimulated hemidiaphragm after 72 h of mechanical ventilation [171]. Another observational study [172] performed unilateral phrenic nerve stimulation in patients undergoing mechanical ventilation during cardiothoracic surgery and reported increased mitochondrial respiration rates in the stimulated hemidiaphragm.

Recent investigations based on a semiblinded, nonrandomized preclinical interventional study test the hypothesis that early phrenic nerve pacing stimulation is a means of increasing diaphragmatic activity during mechanical ventilation [173]. Further research is needed to determine whether phrenic nerve stimulation is effective and feasible as a strategy for preventing VIDD in ICU patients. Phrenic pacing has the potential to provide full ventilatory support for ventilator-dependent patients who have bilateral diaphragmatic paralysis and intact phrenic nerves. Candidates for this treatment method are primarily patients with high cervical cord quadriplegia or patients with central hypoventilation.

Despite technical improvements in phrenic-pacing systems, activation of the diaphragm does not provide sustained, full ventilatory support [174], and evaluation for phrenic pacing should be performed in specialized centers with experience in the technique. Newer methods that use laparoscopic mapping of motor points and intramuscular stimulation of the diaphragm have shown promising results in patients with non-pulmonary diseases [175–179].

As described above, diaphragm pacing is a novel method that can stimulate the diaphragm. This method could be viewed as an effort to support, maintain, and strengthen the diaphragm in patients with weaning failure. It is in these situations that diaphragm pacing (DP) could be proposed in order to remove positive pressure ventilation and restore a more “physiological” breathing obtained through diaphragm contraction [180].

The pathophysiological basis underlying electrical intramuscular DP is seen in experimental studies that have demonstrated that DP increases type 1 slow-twitch fatigue-resistant fibers, thereby reinforcing the resistance of the muscle to fatigue [181]. The process through which an unused muscle progressively reacquires contractile efficiency and resistance to fatigue is called conditioning. This is a well-known process in diaphragm muscles subjected to pacing [182, 183]. It has been known for some time now that such electrical stimulation has an effect on the muscle fiber of patients with spinal cord injuries [184, 185]. These studies support evidence that diaphragm pacing can be used to remodel the composition of the muscle fiber itself.

The use of DP has been proven in canine animal models to provoke the conversion of diaphragm muscle fibers into slow-twitch muscle fibers [186].

These studies portray a clear model of muscle atrophy from disuse and how electrical stimulation (i.e., pacing) can have effects in its own right, independently of any superior voluntary control. The inactivation of a skeletal muscle, due to disuse or direct trauma, seems to lead to the transformation of type I muscles (slow-twitch, fatigue-resistant, oxidative metabolism) into type II muscles (fast twitch glycolytic metabolism) [187, 188].

The two techniques for the implantation of the intramuscular DP system are the thoracic approach, which has been regularly performed with success in a few centers around the world [189], and a laparoscopic approach, which was developed following anatomical studies [190, 191].

Recently, this technique has also been tested as a temporary tool to avoid diaphragmatic amyotrophy in patients requiring MV for whom spontaneous breathing recovery seemed possible. Thus, these two approaches have led to broadened indications not all validated to date. In fact, some controversy exists as the DP system has been accepted in the USA under compassionate use for some non-pulmonary diseases [191], and recently approved in Europe as first medical device for the treatment of VIDD (*CE Mark Approval*). Currently, there are two ongoing randomized clinical trials (ClinicalTrials.gov Identifier: NCT03107949 and NCT03096639) to investigate the safety, effectiveness, and performance of a temporary percutaneous phrenic nerve pacing device. The Lungpacer Diaphragm Pacing Therapy System™ (DPTS, *Lungpacer Medical Inc.*) is a central venous catheter that incorporates pacing electrodes.

The current clinical trials include patients undergoing invasive mechanical ventilation ≥ 7 days and/or have failed two or more spontaneous breathing trials [SBT]. The pacing was performed by stimulating the diaphragm through daily therapy sessions, with the intention of exercising and rehabilitating the diaphragm muscle and with the objective of accelerating the process of liberation from mechanical ventilation. A multicenter, randomized, controlled, and open-label interventional study [192] will carry out (ClinicalTrials.gov Identifier NCT03096639) to investigate the safety and effectiveness of a temporary transvenous diaphragm pacing (TTVDP). The aim is to improve weaning from MV in up to 88 mechanically ventilated adult patients who have failed at least two spontaneous breathing trials over at least 7 days.

Patients will be randomized to TTVDP as the intervention group or standard of care as the control group. The primary endpoint is time to successful extubation with no reintubation within 48 h. Secondary endpoints include physiological parameters such as the maximal inspiratory pressure and ultrasound-measured changes in diaphragm thickness and diaphragm thickening fraction over time. It is expected that enrollment will be completed by September 2019, and this will be the first clinical trial of TTVDP in critically ill patients with difficultly weaning.

Considering the current evidence, there are limitations regarding the effectiveness and safety on the use of DP in critically ill patients with VIDD.

Until more results are available from forthcoming clinical and translational studies in this patient population, this therapy should for the moment be considered as a rescue intervention and always performed in specialized centers with experience.

## Future directions

The majority of critically ill patients admitted to the ICU require mechanical ventilation, and a substantial amount of time spent in the ICU is dedicated to withdrawal from the ventilator [1–3]. It is during the weaning process that diaphragmatic function becomes so important, as it is a major determinant of weaning success and clinical outcomes [192].

The novel concept of myotrauma may have several implications for research and practice [193]. First, future observational studies and clinical trials regarding acute respiratory failure should consider investigating myotrauma as an explanatory mechanism underlying treatment effects.

**Fig. 3:**
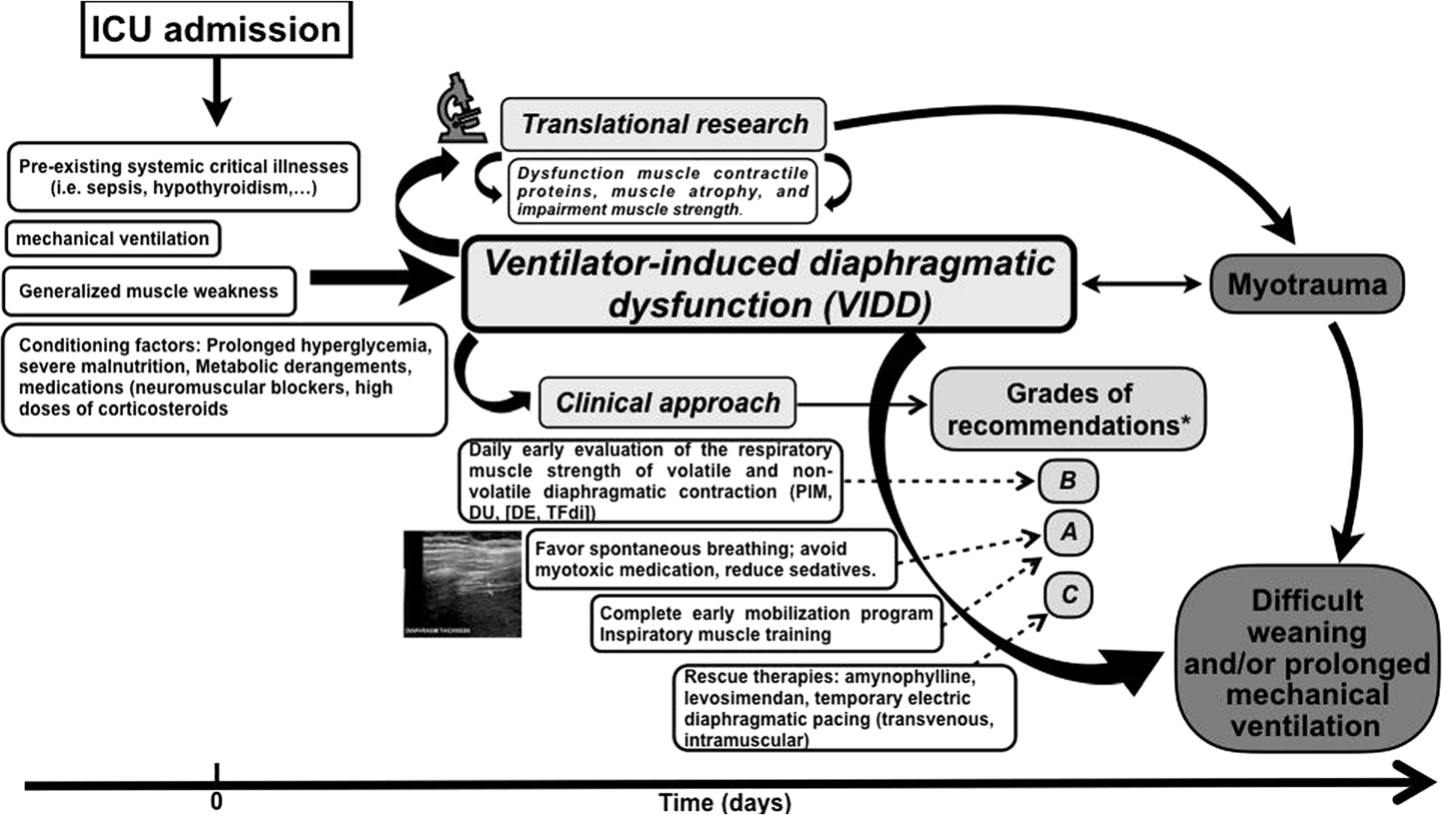
A practical approach for the management of diaphragm dysfunction in critically ill patients. Abbreviations: DU, diaphragmatic ultrasound; DE, diaphragmatic excursion; PIM maximum inspiratory pressure; TFdi, thickening fraction. Asterisk refers to reference [196]

The concept of “bundling” adjunctive therapies is well established in the ICU, and one could even envision the concurrent use of several interventions such as diaphragm pacing with pharmacological interventions to prevent or treat VIDD in the future (Fig. 3). Until further research addresses these clinical challenges, clinicians are left with limited options: to reduce where possible controlled mechanical ventilation through the use of assisted modes and frequent reassessments of the patient’s ability to resume effective spontaneous breathing [194, 195].

While using interventions that might affect patient inspiratory effort or patient–ventilator synchrony—i.e., invasive or non-invasive ventilation strategies, sedation strategies, high-flow oxygen therapy, and extracorporeal life support techniques— the possibility of myotrauma should be considered as a mechanism which may impact on outcomes. The benefits of early mobilization and sedation avoidance strategies might, in part, result from improved recruitment of respiratory muscle effort and avoidance of over-assistance myotrauma.

Ultrasound is a powerful tool used to diagnose VIDD in the clinical setting—it enables assessment of both respiratory effort and detection of structural changes in the muscle and the development of muscle weakness. Future studies should consider incorporating simple diaphragm ultrasound measurements to explore the role of the various forms of myotrauma and diaphragm weakness in the determination of outcomes and hence integrate ultrasound in algorithms for decision-making process.

Concerns about myotrauma are further increased by the possibility that it might contribute to long-term functional disability in ICU survivors.

The major implication of the myotrauma paradigm might be to provide a conceptual framework for how to titrate ventilator support to prevent diaphragm injury.

Evidence suggests that the optimal level of respiratory muscle effort might be that of healthy individuals breathing at rest, equivalent to a respiratory muscle pressure swing of 5–8 cm H_2_O [197]. The aforementioned mediation analyses provide further support for a potential causal relationship between optimal effort and clinical outcome. Importantly, the potential impact of modulating inspiratory effort on major outcomes remains uncertain because of the wide confidence intervals associated with the data regarding effect mediation by diaphragm injury.

Monitoring of respiratory effort should become a routine aspect of clinical practice in the ICU. In view of the current evidence, clinicians should attend to patient inspiratory effort and minimize the duration of diaphragm inactivity during invasive mechanical ventilation. If there is no clear indication for neuromuscular blockade, clinicians should aim to maintain a normal level of spontaneous inspiratory effort as well as minimize the volume and transpulmonary pressure applied to the lung in accordance with the paradigm of protective ventilatory strategy. Indeed, the implementation of a diaphragm-protective ventilation strategy aimed in avoiding changes in diaphragm structure, function and myotrauma should be examined in further clinical translational studies.

## Conclusions

Ventilator-induced diaphragmatic dysfunction is a common deleterious effect of invasive mechanical ventilation and a current relevant clinical challenge. The pathophysiological pathways include several alterations mainly produced by a protein imbalance and oxidative stress. VIDD is often associated with poor clinical outcomes. A thorough evaluation is required to identify its origin and properly manage its effects on symptoms, sleep homeostasis, and exercise capacity.

The increasing availability of diaphragmatic ultrasound has provided a simple and effective means of routinely evaluating diaphragm function at the bedside that should help clinicians identify the optimal treatment for the correct patient.

Referral to a center with experience in this disease and access to diaphragmatic ultrasound, phrenic stimulation or pacing and surgical expertise in diaphragm stimulation should be considered, where applicable.

The integration of evidence from a large body of research based on preclinical and experimental studies of animal models and critically ill patients should allow us to develop a conceptual framework for understanding VIDD. Implementing this knowledge to patient management will eventually result in improved clinical outcomes.

## Data Availability

Data sharing is not applicable to this article as no datasets were generated or analyzed during the current study. Not applicable.

## Abbreviations

ATG5: Autophagy-related 5
BAMPS: Bilateral anterior magnetic phrenic nerve stimulation
CI: Confidence interval
CMV: Controlled mechanical ventilation
CMVC: CMV treated with calpeptin
CON: Control animals
CSA: Cross-sectional area
EAdi: Diaphragm electrical activity
EMG: Electromyography
EMGdi: Diaphragm electromyography
GADPH: Glyceraldehyde-3-phosphate dehydrogenase
ICU: Intensive care unit
MHC: Myosin heavy chain
MIP: Maximal inspiratory pressure
MOS: Mitochondrial oxidative stress
MV: Mechanical ventilation
PdiTw: Transdiaphragmatic twitch pressure
PEEP: Positive end-expiratory pressure
PW-TDI: Pulse wave tissue Doppler imaging
ROS: Reactive oxygen species
TTVDP: Temporary transvenous diaphragm pacing
UPP: Ubiquitin–proteasome pathway
VIDD: Ventilator-induced diaphragm dysfunction
VILI: Ventilator-induced lung injury
VT: Tidal volume
